# Non adherence to inhalational medications and associated factors among patients with asthma in a referral hospital in Ethiopia, using validated tool TAI

**DOI:** 10.1186/s40733-017-0035-0

**Published:** 2017-10-06

**Authors:** Asnakew Achaw Ayele, Henok Getachew Tegegn

**Affiliations:** Department of Clinical Pharmacy, School of Pharmacy, College of Medicine and Health Sciences, University of Gondar, P.O.Box: 196, Gondar, Ethiopia

**Keywords:** Non adherence, Inhalational, Asthma, Gondar, Ethiopia

## Abstract

**Background:**

Asthma is a chronic inflammatory condition of the airways that affects roughly 358 million people globally. It is a serious global health problem with an increasing prevalence worldwide. Most people affected are in low- and middle-income countries including Ethiopia. The association between non -adherence and poor disease control is clearly stated in different literatures. The main objective of the present study was to assess self-reported non- adherence level and to identify the potential factors associated with non-adherence.

**Methods:**

An institution based cross-sectional study was conducted in university of Gondar teaching and referral hospital. The data was collected using a validated tool called Test of Adherence to Inhalers (TAI).

**Result:**

Among the total of study participants, higher proportions of patients were female (57.3%). Large number of the respondents (59.1%) were Unable to read and write. 18.3% of inhalational user asthmatic patients were not adherent to inhalational medications. According to this study only 49.4% of the respondents were adherent to inhalations and 32.3% of them were intermediate adherent to inhalational anti asthmatics medications. Lack of education about the Proper use of inhalational anti-asthmatics medications, poly pharmacy and co-morbidities were statistically significant factors associated with non-adherence.

**Conclusion:**

The rate of non-adherence to inhalational anti asthmatics is high. Therefore, promoting optimal medication adherences through education, proper patient consultation is essential to optimize the benefits of treatment. Measurement of the degree of non-adherence to inhaled treatment in each individual patient is important in early interventional practice.

## Background

Asthma is a chronic inflammatory condition of the airways that affects roughly 358 million people [1]. It is a serious global health problem with an increasing prevalence worldwide. Most people affected are in low- and middle-income countries, and its prevalence is estimated to be increasing fastest in those countries. People of all ages are affected by this chronic airways disorder with higher burden of disability [2]. In 2015, more than 397,100 deaths is caused by asthma and most of which occurred in the developing world [3]. In Ethiopia Asthma deaths reached 0.89% of total deaths. Asthma is thought to be caused by a combination of both genetic and environmental factors. Symptoms can be prevented by avoiding triggers, such as allergens and irritants. While there is no cure for asthma, symptoms can be improved with combination of medications and avoiding triggering factors. Short-acting beta_2_ agonists are the first line treatment for acute asthma symptoms and anticholinergic medications provide additional benefit when used in combination with Short acting beta2 agonists [4]. Currently corticosteroids are the most effective treatment available for long-term control of asthma [5]. Except in the case of severe persistent disease, I-inhaled forms of corticosteroids are usually used in the long term management [5]. Good level of adherence is the cornerstone in the long term management of asthma. In chronic asthmatic patients, non-adherence or inhaler mishandling increases mortality, morbidity, and hospital admission [6, 7].

The association between non -adherence and poor disease control is clearly stated in different literatures. Patient age, level of education/knowledge about the conditions, medication and lack of technical skill of delivery devices were taught to be the cause of non-adherence to inhaled medications [8–11]. In addition to this, complexity of the inhalation regimen, peculiarities of inhaler devices, type of inhaled agent, and a variety of patient beliefs and sociocultural and psychological factors also the cause poor adherence to inhalational medications. [12]. It is important to know that the rational use of inhaled medication improves the outcomes of chronic asthma managements. Among patients receiving long-term pharmacotherapy for chronic diseases, only 50% of them are adherent to treatment while adherence rates in asthma have been shown to vary widely from 22% to 78% [13–16]. Different studies have been published regarding in measuring of non-adherence rate in inhaled medications using standardized Patient self-completed questionnaires. Because they are not adequately validated, they become nonspecific for inhaled medications which results in invariably underestimates the incidence of non-adherence rates. The main aim of the present study was to assess self-reported non adherence level and to identify the potential factors associated with nonadherence, using a validated tool called Test of Adherence to Inhalers (TAI) [17].

## Methods study design and setting

An institution based cross-sectional study was conducted in university of Gondar teaching and referral hospital (UoGTRH) from September 2016 to December 2016.

## Data collection instrument

The data was collected using a tool called Test of Adherence to Inhalers (TAI), which is validated in 2016 by PLAZA et al. [17] to assess the level of adherence to inhalers in asthma and COPD patients. TAI consists of both 10 and 12 item questions. The 10- items TAI was designed to identify non-adherent patients and to measure the non-adherence level, whereas the 12-items TAI was designed to guide clinically the non-adherence patterns. TAI scores 50, 46–49 and less than 45 was considered as adherent, intermediate adherent and non-adherent respectively. In the present study the 10 item TAI is used to measure the level of non-adherence. In addition to TAI tool, patients demographics and clinical data was collected. Poly pharmacy is operationally defined as-patients who are taking more than four drugs and co-morbidity is considered if a patients have more than 1 confirmed diagnosis. The questionnaires were initially translated to Amharic (national language of Ethiopia) and then back to English in order to ensure that the translated version gives proper meaning.

### Patients’ recruitment

Patients greater than 18 years of age and those who uses inhaled medications for at least 2 years before enrolment were included in the study. Study participants were consecutively enrolled from September 2016 to December, 2016 when they visit Gondar university hospital outpatient pharmacy for refill. The data was collected by pharmacists who have been working in the outpatient pharmacy. TAI is self-compiled questionnaire but for patients who are unable to read and write, the data collectors helped them into the completion of the questionnaire through interview.

## Data entry and analysis

The data was entered to and analyzed using IBM SPSS Statistics for Windows, version 20. Continuous variables were presented as mean ± standard deviation. Categorical variables were expressed as frequencies and percentages. Association between predictive variables (sociodemographic and clinical data of patients) and dependent variables (non -adherence) using binary logistic regression was done to identify determinants of non-adherence. Variable with *p* value <0.20 were included in the multivariate model and *P*-value less than 0.05 and 95%, confidence interval (CI) were used as cut off points for determining statistical significance of associations among different variables.

## Ethical consideration

Ethical approval was obtained from research and ethics review committee of school of Pharmacy, University of Gondar to conduct the study. Informed verbal consent was also obtained from each respondent after explaining the purpose of the study. They were also informed that participation was voluntary and they could withdraw from the study at any stage if they desired. Participant’s confidentiality was guaranteed by not recording their personal identifiers on the questionnaire.

## Result

### Socio demographics and clinical data of the participants

A total of 164 asthmatic patients who meets the inclusion criteria were included in the study. Among the total of study participants, higher proportions of patients were female (57.3%) (Table 1). Most of the participants (47%) were in between 18 and 30 age group. Large number of the respondents, 97 (59.1%) were unable to read and write. A total of 18.9% patients had previous history of adverse drug reaction (ADR) to inhalational anti asthmatic medications. The mean (±standard deviation) duration of years living with the disease was 6.09 ± 3.13 ranging from 2 to 19 years. Respondents who were not taking inhaler education was found to be 18.9%, whereas 6.7% were smokers as shown in Table 1.

**Table 1:** Socio-demographic characteristics of the respondent-UOGTRH, 2016

Patient characteristics and clinical data	N (%)
Total number of study population, N	164
Sex	
Male, n (%)	70 (42.7)
Female, n (%)	94 (57.3)
Age	
18–30, n (%)	(77, 47)
31–45, n (%)	(56, 34)
46–64, n (%)	(18, 11)
> 65, n (%)	(13, 7.9)
Education level	
Unable to read and write, n (%)	97 59.1
Primary education, n (%)	31 18.9
Secondary education n (%)	29 17.7
Higher education, n (%)	7 4.3
Patient’s clinical data, n (%)	
Previous history of ADR	
Yes, n (%)	31 (18.9)
No, n (%)	133 (81.1)
Duration of disease, years mean (SD)	6.09 + 3.13
Previous inhaler education	
Yes, n (%)	52 (81.1)
No, n (%)	112 (19)
Smoking history	
Yes, n (%)	11 (6.7)
No, n (%)	153 (93.3)
Poly pharmacy	
Present	47 (28.6)
Absent	117 (71.4)
Co-morbidity	
Yes	64 (39.1)
No	100 (60.9)
Adherence level	
Adherent (TAI score≥50), n (%)	81 (49.4)
Intermediate adherent (TAI score 46–49), n (%)	53 (32.3)
Non-adherent (TAI score ≤ 45), n (%)	30 (18.3)

Among the total participants, ploy pharmacy was present in 28.6% of them. Regarding to co-morbidity, 39.1% of the participants has had at least one more disease in addition to asthma. Table 1 Details of sociodemographic data of the participants.

### Non adherence level to inhalational anti- asthmatics medications

The level of adherence to inhalers among the participants was assessed using 10- items TAI tool. According to this study 49.4% of the respondents were adherent to inhalations with TAI score greater than 50 and 32.3% of them (TAI score 46–49) were intermediate adherent to inhalational anti asthmatics medications. However the result of this study reveals that 18.3% of asthmatic patients (TAI score ≤ 45) were not adherent to inhalational anti asthmatics medications. Response of the TAI questions of the respondents is summarized in Table 2.

**Table 2:** Response of the respondents to TAI questionnaires- UOGTRH, 2016

TAI questions	TAI scores				
	All n (%)	More than half n (%)	About half n (%)	Less than half n (%)	None n (%)
How many times did you forget to take your regular inhalers in the last 7 days?	6 (3.7)	11 (6.7)	13 (7.9)	18 (11.6)	115 (70.1)
	Always n (%)	Almost always n (%)	Sometimes n (%)	Almost never n (%)	Never n (%)
You forget to take your inhalers:	7 (4.3)	10 (6.1)	11 (6.7)	15 (9.1)	121 (73.8)
When you are feeling well, you stop taking your inhalers:	7 (4.3)	13 (7.9)	7 (4.3)	34 (20.7)	103 (62.8)
At the weekend or when you go on holiday, you stop taking your inhalers:	12 (7.3)	8 (4.9)	5 (3)	18 (11)	121 (73.8)
When you are anxious or sad, you stop taking your inhalers:	4 (2.4)	5 (3)	12 (7.3)	13 (7.9)	130 (79.3)
You don’t take your inhalers out of fear of potential side effects	7 (4.3)	8 (4.9)	15 (9.1)	2 (1.2)	132 (80.5)
You stop taking your inhalers because you believe that they are of little help in treating your disease:	3 (1.8)	9 (5.5)	14 (8.5)	38 (13.2)	100 (61)
You take fewer inhalations than prescribed by your doctor:	6 (3.7)	6 (3.7)	16 (9.8)	39 (23.8)	97 (59.1)
You stop taking your inhalers because you believe that they interfere with your day-to-day or work life:	4 (2.4)	7 (4.3)	11 (6.7)	44 (26.8)	98 (59.8)
You stop taking your inhalers because you have trouble paying for them:	11 (6.7)	6 (3.7)	7 (4.3)	26 (15.9)	114 (69.5)

In Multivariate logistic regression analysis, respondents who had not received education about inhalational use were about 8 times more likely to be non-adherent compared to those who had received(AOR = 8.14, 95% CI, 1.27, 47.29)(Table 3). Respondents who have experienced ADR to inhalational anti-asthmatics previously were more likely to be non-adherent. The presence of co-morbidity was associated with increased non-adherence to inhalational medications (AOR = 4.60 95% CI, 2.39, 12.945). The occurrence of non-adherence to inhalational medications was 6 times more likely among patients with poly pharmacy (AOR = 6.488(95%CI, 3.31, 15.77). Patients who had no formal education were 2.71 times more likely to being non adherent to their medications than those who attended higher education (AOR = 2.71 (95%CI, 1.62, 7.66). However other characteristics (age group and sex) were not significantly associated with non-adherence to inhalational medications.

**Table 3:** Patient’s clinical and demographic data associated with non-adherence-

Variables	Non - adherence		OR (95% CI)		*P*-value
	Yes	No	COR	AOR	
Age					
18–30	5	72	1	1	–
31–45	6	50	0.769 (0.39–1.52)	0.83 (0.35, 1.97)	0.671
46–64	7	11	0.79 (0.27–2.30)	1.39 (0.20–9.56)	0.545
> 65	10	3	1.24 (0.28–5.34)	1.38 (0.18–10.22)	0.432
Education level					
Unable to read and write	23	74	1.8 (0.92–3.24)	2.71 (1.62, 7.66)^a^	0.032
Primary education	7	24	2.48 (0.37–18.25)	0.08 (0.10–0.79)	0.553
Secondary education	4	25	0.66 (0.11–3.84)	0.38 (0.03–4.37)	0.653
Higher education	2	5	1	1	–
Patients clinical data					
Previous inhaler education					
Yes	5	49	1	1	–
No	64	48	2.24 (0.59–8.37)	8.14 (1.27,47.29)^a^	0.002
Previous history of severe ADR					
Yes	20	11	6.67 (0.61–73.03)	29.87 (1.26, 708.6)^a^	0.035
No	26	107	1	1	_
Poly pharmacy					
Present	39	8	2.09 (0.88–4.30)	6.488 (3.310, 15.770)^a^	0.027
Absent	28	89	1	1	–
Co-morbidity					
Yes	44	20	0.76 (0.55, 1.03)	4.60 (2.39, 12.945)^a^	0.026
No	23	77	1	1	–

*COR* crude odds ratio, *AOR* adjusted odds ratio, *ADR* adverse drug reactions

^a^-statically significant

## Discussion

To our best knowledge, non-adherence of asthmatic patients to inhalational medications was scarcely investigated in Ethiopia. Although there are few published studies, and they have used different methods to assess non adherence of asthmatic patients to treatment, these data lacks the methodology specific to inhalational ant-asthmatics. This study was held in order to assess the level of non-adherence and to identify factors associated with non-adherence using TAI tool which is specific to inhalational medications. The result of the present study reveals that 49.4% of the asthmatic patients were adherent and 32.3% were intermediate adherent to their medications. However the prevalence of non-adherence is 18.3%. This percentage shows that still many asthmatic patients are not adherent to their inhalational medications. Previously done research shows that, adherence to inhaled therapies is worse than that seen with oral or injected therapies in patients with asthma in different age groups [18–21].

According to study conducted in turkey to assess the effects of training on the correct use of inhalation devices, only 55.3% of patients were able to correctly use the devices before training but after the training, the rate of correct use increased to 83.7% [22]. Valid educational programs for asthmatics can improve the knowledge about the disease and to understand how they take their inhalational medications to improve adherence. Patients attending two lessons with helpful training tools significantly increase their knowledge about asthma treatment compliance and patient self-management [23].

Identifying the reasons for non-adherence to inhalational medications is essential in order to determine the best way to intervene and to increase the control of asthma. The result of previously published study shows that age of the patients, knowledge about the disease, lack of skills about delivery devices, regimen complexity of the medication regimen, adverse effects And lack of health education are some of the factors that affects adherence to inhalational medications [24–29]. Higher rates of non-adherence been associated with Inhaler use potentially because of the increased complexity introduced by the inhalers and lack of skills for appropriate use [30, 31]. In our study from a total of patients who had not received education about proper inhalational medication use, 57% of them are not adherent to their medications. Moreover 18.9% of the study participants did never receive any instruction to inhaler technique from health care givers and medication dispensers. The Odds of non-adherence for a subject who had not received education about proper inhalational medication use had a very high probability of being non adherent when compared to a subject who had received education (AOR = 8.14; 95%CI: 1.27, 47.29).

Result of previous study shows that the major nondrug factors associated with non-adherence is fear about side effects to the medications [26]. The finding of this study reveals that patients with experience of previous ADR were more likely to be non-adherent to their medications. This is due to the fact that patients may not take their medications, if they fear side effects. Efforts to minimize the level of non-adherence to inhalational medications should be made by letting patients know at the start of the treatment which side effects are possible with a given regimen, monitoring for such effects and provide treatment for adverse effects. This can be achieved through pharmacists even from beginning with the first dispensing of inhalational anti asthmatics. Another important issue regarding the factors that affect non-adherence to inhalational medications is that poly pharmacy. In the present study Patients who have taken an average of more than 4 drugs are more likely to become non-adherent. Co-morbidity is also one factor that have negative impact on the outcomes of asthma treatment. In our study patients with co-morbidity is 4.6 is more likely to be non-adherent.

### Limitations of the study

This study tried to assess the level of non- adherence using a self-reported survey, which subjects it to the recall and social desirability biases. The other limitation was that types of ADR, specific comorbidities and types inhalation medications patients had is not addressed. The sample size obtained during the study period is rather small as it has been done in single centered setup.

## Conclusion

The rate of non-adherence to inhalational anti-asthmatics is high. Lack of education about the Proper use of inhalational anti-asthmatics medications, poly pharmacy and co-morbidities have been identified to have affected non-adherence rate. Therefore, promoting optimal medication adherences through education, proper patient consultation essential to optimize the benefits of treatment. Subsequently, measurement of the degree of non-adherence to inhaled treatment in each individual patient becomes increasingly important in early interventional practice.
